# A severe shortage of hyperbaric units in Egypt: a call to fill this shortage

**DOI:** 10.1007/s43999-022-00015-1

**Published:** 2022-12-07

**Authors:** Ali Mohamed Ali Ismail

**Affiliations:** Department of Physical Therapy for Cardiovascular/Respiratory Disorder and Geriatrics, Faculty of Physical Therapy, Cairo University, Giza, Egypt

## Dear editor

Hyperbaric oxygen therapy (HBOT) is defined as inhaling 100% oxygen at a pressure greater than that of sea level, or at one atmosphere above absolute zero (ATA). A pressure of at least 1.4 ATA is necessary to have a clinical effect, according to the Underwater and Hyperbaric Medical Society (UHMS) [1]. In monoplace or multiplace chambers, the safe and economical therapy, HBOT, is often administered daily for several weeks (i.e. accommodating a single patient or several) [2]. By increasing the partial pressure of oxygen in all tissues, hyperbaric oxygen may reduce swelling, activate fibroblasts and macrophages, and increase angiogenesis and collagen formation [3].

Several recognized indications for the treatment include air/gas embolism, gas gangrene, carbon monoxide poisoning, compartment syndrome, severe anemia, arterial occlusion/insufficiency, compromised flaps/grafts for wound healing, refractory osteomyelitis, different necrotizing soft-tissue infectious diseases, acute thermal burn injuries, intracranial abscess, idiopathic sudden sensorineural hearing loss, delayed radiation injury, and diving decompression [2].

Comparing the cost of hyperbaric oxygen therapy with the cost of conservative treatments for some disease conditions has been previously studied. It has been proven that hyperbaric oxygen therapy reduces the amount of time and money spent in treating these conditions more than conservative treatments.

In a Canadian trial, the cost-effectiveness of supplemental HBOT versus standard care alone in treating diabetic foot ulcers was compared. In comparison to CND$49,786 for standard care alone, the 12-year cost for those getting HBOT was CND$40,695. The results were 3.64 quality-adjusted life years (QALYs) for HBOT recipients against 3.01 QALYs for controls. It was estimated that it would cost CND$14.4–19.7 million over the course of four years to treat all cases of common diabetic foot ulcers in Canada. Nineteen to thirty-five additional machines would be needed nationwide if seven-person HBOT chambers were deployed [4].

Despite the importance and cost-effectiveness of HBOT in many medical fields, there is a severe shortage in the number of governmental HBOT units in Egypt. At the level of 29 Egyptian governorates, there are only 4 governmental HBOT units in Egypt: a unit at the Nasser Educational Institute affiliated to the Egyptian Ministry of Health, a unit at Aeromedical Council Hospital (Aviation Institute) affiliated to the Ministry of Civil Aviation, a unit at the Specialized Air Hospital affiliated with the Egyptian Armed Forces, and a unit at the Center for Children with Special Needs affiliated to the Faculty of Graduate Studies for Childhood at Ain Shams University. What is noteworthy in the matter is the presence of the four governmental HBOT units in Cairo governorate, without the other 28 governorates. The cost of one hour-long session ranges between 100-300 Egyptian pounds (5.29–15.87 USD) in public-hospital HBOT units.

As for the private HBOT units or centers in Egyptian governorates, there is one unit in Giza (El-Hadaray center), one unit in Cairo (Dr. Hani Khalil center), two units in Red Sea governorate (OxyMed center and the dolphin autism center in Hurghada city), and one unit in Menoufia and Assiut governorates. The cost of one hour-long session ranges between 500-800 Egyptian pounds (26.45–42.32 USD) in private HBOT units.

When comparing the price of the governmental HBOT session with the private one, the governmental HBOT session, despite its somewhat high price, which is not commensurate with the monthly income (2400–9000 Egyptian pounds or 126.98-476.19 USD) of the vast segment of Egyptians - is cheaper than the private HBOT session. Most of the time, this may create a waiting HBOT list of up to 6 months in governmental HBOT units.

The high price of private HBOT sessions forces low-income patients (the mean monthly income of those population is 2400 Egyptian pounds or 126.98USD) – who represent a very large percentage of the Egyptian population - to move from all governorates to the Egyptian capital, where the price of a governmental HBOT session is somewhat cheap. Severe crowding, high and continuous demand, long waiting lists, most patients, especially those who do not reside in Cairo governorate, are forced not to start or continue HBOT sessions, which most of the time need to be taken daily (one or two sessions per the day) and for a number that may range from forty to eighty sessions per patient as in some fungal infections including mucormycosis [5].

Hospitals affiliated with the Ministry of Health and/or Higher Education should pay attention to this severe shortage of HBOT units and allocate a part of their financial budget to equip HBOT units in all governorates of Egypt, especially governorates far from Cairo.

## Data Availability

Not applicable.
